# A Likelihood-Free Estimator of Population Structure Bridging Admixture Models and Principal Components Analysis

**DOI:** 10.1534/genetics.119.302159

**Published:** 2019-04-26

**Authors:** Irineo Cabreros, John D. Storey

**Affiliations:** * Program in Applied and Computational Mathematics, Princeton University, New Jersey 08544; † Lewis-Sigler Institute for Integrative Genomics, Princeton University, New Jersey 08544

**Keywords:** admixture, genetic structure, logistic factor analysis, method of moments, nonparametric, PCA, population stratification, population structure, unifying

## Abstract

We introduce a simple and computationally efficient method for fitting the admixture model of genetic population structure, called ALStructure. The strategy of ALStructure is to first estimate the low-dimensional linear subspace of the population admixture components, and then search for a model within this subspace that is consistent with the admixture model’s natural probabilistic constraints. Central to this strategy is the observation that all models belonging to this constrained space of solutions are risk-minimizing and have equal likelihood, rendering any additional optimization unnecessary. The low-dimensional linear subspace is estimated through a recently introduced principal components analysis method that is appropriate for genotype data, thereby providing a solution that has both principal components and probabilistic admixture interpretations. Our approach differs fundamentally from other existing methods for estimating admixture, which aim to fit the admixture model directly by searching for parameters that maximize the likelihood function or the posterior probability. We observe that ALStructure typically outperforms existing methods both in accuracy and computational speed under a wide array of simulated and real human genotype datasets. Throughout this work, we emphasize that the admixture model is a special case of a much broader class of models for which algorithms similar to ALStructure may be successfully employed.

## Abstract (precis)

Characterizing genetic variation in humans is an important task in statistical genetics, enabling disease-gene mapping in genome-wide association studies (GWAS) and informing studies of human evolutionary history. A common approach to quantifying genetic variation...

UNDERSTANDING structured genetic variation in human populations remains a foundational problem in modern genetics. Such an understanding allows researchers to correct for population structure in GWAS studies, enabling accurate disease-gene mapping (Knowler *et al.* 1988; Marchini *et al.* 2004; Song *et al.* 2015). Additionally, characterizing genetic variation is important for the study of human evolutionary history (Cavalli-Sforza *et al.* 1988; Esteban *et al.* 1998; Li *et al.* 2008).

To this end, much work has been done to develop methods to estimate what Alexander *et al.* (2009) term *global ancestry*. In the global ancestry framework, the goal is to simultaneously estimate two quantities:

- the allele frequencies of ancestral populations.
- the admixture proportions of each modern individual.

Many popular global ancestry estimation methods have been developed within a probabilistic framework. In these methods, which we will refer to as *likelihood-based* approaches, the strategy is to fit a probabilistic model to the observed genome-wide genotype data by either maximizing the likelihood function (Tang *et al.* 2005; Alexander *et al.* 2009) or the posterior probability (Pritchard *et al.* 2000; Raj *et al.* 2014; Gopalan *et al.* 2016). The probabilistic model fit in each of these cases is the *admixture model*, described in detail in the *Model and Theory* section below, in which the global ancestry quantities (i) and (ii) are explicit parameters to be estimated.

A related line of work relies on principal components analysis (PCA) and other eigen-decomposition methods, rather than directly fitting probabilistic models; as such, we will refer to them collectively as *PCA-based* approaches. These methods find many of the same applications as global ancestry estimates while obviating a direct computation of global ancestry itself. For example, the EIGENSTRAT method of Patterson *et al.* (2006) and Price *et al.* (2006) uses the principal components of observed data to correct for population stratification in GWAS, avoiding altogether the estimation of admixture proportions or ancestral allele frequencies. Similarly, Hao *et al.* (2016) observe that many important applications of global ancestry really only require *individual-specific allele frequencies*. In a sense, individual-specific allele frequencies are simpler than global ancestry; while global ancestry specifies all of the individual-specific allele frequencies, the converse is not true. Therefore, Hao *et al.* (2016) introduce a simple truncated-PCA method that accurately and efficiently estimates individual-specific allele frequencies alone.

Both likelihood-based and PCA-based approaches have distinct merits and drawbacks. The PCA-based methods are computationally efficient and accurate in practice. It is shown, for instance, that the individual-specific allele frequencies obtained by truncated-PCA are empirically more accurate than those obtained by likelihood-based methods (Hao *et al.* 2016). Another attractive feature of PCA-based methods is that they make minimal assumptions about the underlying data-generative model. However, as mentioned before, PCA-based methods do not provide the full global ancestry estimates that their corresponding likelihood-based methods do. Most notably, they do not provide direct estimates of admixture proportions, which are often of primary interest in some applications. Additionally, PCA-based methods often have weaker statistical justifications, as they are typically not based on a probabilistic model. Although Tipping and Bishop (1999) introduced a probabilistic interpretation of PCA for multivariate Normal data, to our knowledge, no such interpretation of PCA exists when the data are Binomial, as is the case in the admixture model.

Recognizing the relative advantages of each approach, several researchers have attempted to bridge the gap between likelihood-based and PCA-based approaches. In spirit, this is also the approach that we take in the present work, and so we briefly review previous contributions to contextualize the advances made by our own method. Engelhardt and Stephens (2010) observed that fitting the admixture model was related to PCA in the sense that both could be posed as matrix factorization problems, which differ only in the constraints imposed on factors. They then introduced a third matrix factorization problem, called Sparse Factor Analysis (SFA), which encourages a sparsity through a particular prior. However, since SFA does not enforce the probabilistic constraints of the admixture model (nor the orthogonality constraints of PCA), its output cannot be directly interpreted as an estimate of global ancestry. Lawson *et al.* (2012) provided further insight into the mathematical relationship between admixture models and PCA and introduced a method for the analysis of phased haplotype data. This method, called fineSTRUCTURE, fits a version of the admixture model in which each observed individual belongs to a single (rather than admixed) population. Zheng and Weir (2016) introduced a method called EIGMIX that leverages PCA to infer admixture proportions from unphased genotype data. While EIGMIX allows individual genomes to be derived from a mixture of multiple ancestral populations (unlike fineSTRUCTURE), it requires a set of sampled individuals known to be derived from single ancestral populations. A related line of work uses PCA-based approaches to fit models of *local ancestry*, in which inferences about the ancestry of individual genetic loci are desired (for example, Brisbin *et al.* 2012).

While the aforementioned literature illustrates that PCA can be leveraged to provide information about population structure, each approach falls short of providing complete estimates of global ancestry under the general admixture model. The method which we introduce in the present work, called ALStructure, does precisely this. ALStructure requires no additional assumptions (such as the existence of unadmixed individuals in Zheng and Weir 2016), no specialized input (such as the unphased haplotypes of Lawson *et al.* 2012), and provides direct estimates of admixture proportions (unlike Engelhardt and Stephens 2010). As such, ALStructure is the only existing PCA-based method that can provide a direct substitute to the most popular likelihood-based approaches. As an additional important advantage, the underlying mathematical theory that justifies ALStructure is sufficiently general so as to apply to a class of models that subsumes the admixture model. As such, we believe that imitable algorithms to ALStructure could be useful beyond the present genetics application.

The basic strategy of ALStructure is to eliminate the primary shortcomings of PCA-based methods while retaining their important advantages over likelihood-based methods. In particular, we extend the approach taken in Hao *et al.* (2016) in two ways. First, we replace classical PCA with the closely related method of *Latent Subspace Estimation* (LSE) (Chen and Storey 2015). In so doing, we will make mathematically rigorous the empirically effective truncated-PCA method of Hao *et al.* (2016) for estimating individual-specific allele frequencies. Second, we use the method of *alternating least squares* (ALS) to transform the individual-specific allele frequencies obtained via LSE into estimates of global ancestry.

We perform a body of simulations and analyze several globally and locally sampled human studies to demonstrate the performance of the proposed method, showing that ALStructure typically outperforms existing methods both in terms of accuracy and speed. We also discuss its implementation and the trade-offs between theoretical guarantees and run-time. We find that ALStructure is a computationally efficient and statistically accurate method for modeling admixture and decomposing systematic variation due to population structure.

The remainder of this paper is organized as follows. *Model and Theory* introduces the admixture model and details the mathematical underpinnings of our approach. *The ALStructure Algorithm* summarizes the ALStructure algorithm. A reader primarily interested in a basic understanding of the operational procedure of ALStructure and its applications may proceed to *The ALStructure Algorithm* after reading *The admixture model* subsection, as the remainder of *Model and Theory* is more technical in nature. *Results From Simulated Data* and *Applications to Global Human Studies* assess the performance of ALStructure on a wide range of real simulated datasets.

## Model and Theory

In this section and the following we present the ALStructure method and detail some of its mathematical underpinnings. In *The admixture model*, we define the *admixture model*: the underlying probabilistic model assumed by ALStructure. *Optimal model constraints* describes the overall strategy of ALStructure as an optimality search subject to constraints rather than navigating a complex likelihood surface. *Leveraging constraints to estimate $\hat{F}$* describes how the constraints can be used to estimate individual-specific allele frequencies. In *Latent subspace estimation* we present a mathematical result from Chen and Storey (2015) upon which the ALStructure algorithm heavily relies. *Leveraging constraints to estimate P and Q* describes why estimating global ancestry, given the individual-specific allele frequencies, is equivalent to a constrained matrix factorization problem. An efficient algorithm based on the method of ALS is also provided in this section for performing the constrained matrix factorization. The complete ALStructure algorithm is then presented in *The ALStructure Algorithm*.

Throughout this work, we adhere to the following notational convention: for a matrix A, we denote the *i* row vector of A by $a_{i\cdot }$, the *j* column vector of A by $a_{\cdot j}$, and the (i,j) element of A by $a_{ij}$.

### The admixture model

The observed data X is an m×n matrix in which *m* (the number of SNPs) is typically much larger than *n* (the number of individuals). An element $x_{ij}$ of X takes values 0, 1, or 2 according to the number of reference alleles in the genotype at locus *i* for individual *j*.

ALStructure makes the assumption common to all likelihood-based methods that the data are generated from the *admixture model*. Under this model, the genotypes are generated independently according to $x_{ij}|f_{ij}∼\text{Binomial}\left(2,f_{ij}\right)$, where F is an m×n matrix encoding all of the Binomial parameters. Each element $f_{ij}$ is an *individual-specific allele frequency*: the frequency of allele *i* in individual *j*. F is further assumed to be of rank *d*, where d≪n≪m. *d* may be thought of as the number of ancestral populations from which the observed population is derived. F then admits a factorization F=PQ, in which P and Q have the following properties:

$$P\in {\mathbb{R}}^{m\times d}\;\text{with}\;p_{ij}\in \left[0,1\right]\;\forall \left(i,j\right)$$

$$Q\in {\mathbb{R}}^{d\times n}\;\text{with}\;q_{ij}\geq 0\forall \left(i,j\right)\;\text{and}\;\sum_{i}q_{ij}=1\forall j$$

The matrices P and Q have the following interpretations: (i) each row $p_{i\cdot }$ of P represents the frequencies of a single SNP for each of the *d* ancestral populations, and (ii) each column $q_{\cdot j}$ of Q represents the admixture proportions of a single individual. Together, P and Q encode the global ancestry parameters of the observed population; the goal of existing likelihood-based methods is to estimate these matrices. By contrast, the truncated-PCA method of Hao *et al.* (2016) is focused on estimating F and not its factors. Equation 1 summarizes the admixture model.

$$\underset{m\times n}{\left(\begin{array}{ccccc} & & F & & \end{array}\right)}=\underset{m\times d}{\left(\begin{array}{ccc} & P & \end{array}\right)}\underset{d\times n}{\left(\begin{array}{ccccc} & & Q & & \end{array}\right)}$$ (1)

The model introduced in Pritchard *et al.* (2000), which we refer to as the *PSD model*, is an important special case of the admixture model. It additionally assumes the following prior distributions on P and Q:

$$p_{ij}∼\text{Balding-Nichols}\left(F_{i},p_{i}\right)$$

$$q_{\cdot j}∼\text{Dirichlet}\left(\alpha \right)$$

The Balding-Nichols distribution (Balding and Nichols 1995) is a reparameterization of the Beta distribution, in which $F_{i}$ is the $F_{ST}$ (Weir and Cockerham 1984) at locus *i*, and $p_{i}$ is the population minor allele frequency at locus *i*. Specifically, $\text{Balding-Nichols}\left(F,p\right)=\text{Beta}\left(\frac{1-F}{F}p,\frac{1-F}{F}\left(1-p\right)\right)$. (Other prior distributions can be used for P and Q (Pritchard *et al.* 2000), but here we refer to the PSD model as that using the priors listed here.) Existing Bayesian methods (Pritchard *et al.* 2000; Raj *et al.* 2014; Gopalan *et al.* 2016) fit the PSD model specifically, while existing maximum likelihood (ML) methods (Tang *et al.* 2005; Alexander *et al.* 2009) and ALStructure require only the admixture model assumptions.

Although we focus on fitting the admixture model in the present work, it is important to note that the general strategy of the ALStructure algorithm is insensitive to the particular details of this model. The necessary features that the theoretical underpinnings of ALStructure require are: (i) higher moments of $x_{ij}$ are bounded, (ii) F is low rank, and (iii) m≫n. (For a precise statement of the theoretical assumptions of LSE, see Chen and Storey 2015.) For example, an imitable algorithm could be applied to high dimensional data X where $x_{ij}|f_{ij}∼\text{Poisson}\left(f_{ij}\right),$ and F is a low rank matrix whose factors P and Q potentially have natural constraints. Because of its generality, we hope that the approach of ALStructure will find useful application beyond the analysis of admixture.

### Optimal model constraints

Most existing methods for fitting the admixture model employ various optimization techniques to search for the ML parameters (Pritchard *et al.* 2000; Alexander *et al.* 2009) or the maximum *a posteriori* estimate (Raj *et al.* 2014; Gopalan *et al.* 2016). Our approach has a fundamentally different character: rather than searching through a rough likelihood landscape in pursuit of an optimal solution, ALStructure seeks a feasible solution to a set of optimal constraints. To be more specific, we begin with the observation that any solution satisfying a particular set of constraints is risk-minimizing among a class of unbiased estimators. Because any feasible solution is optimal, we can think of the constraints themselves as being optimal. Notably, the need to maximize likelihood is altogether obviated.

The challenge of this approach is twofold. First, the constraints themselves need to be estimated from the data: they are not directly observable. This is done through the method of LSE detailed in *Latent subspace estimation*. Second, feasible solutions to the estimated constraints will not typically exist. For this reason, we seek solutions that approximately satisfy the constraints, thereby converting a feasibility problem to a least squares (LS) optimization problem. This procedure is done through the method of ALS and is detailed in *Leveraging constraints to estimate P and Q*. Throughout the remainder of the present subsection, we detail the constraints themselves.

There are several constraints that any reasonable estimate of the parameters of the admixture model must obey. The first is simply that the parameter estimates $\hat{F},$ $\hat{P},$ and $\hat{Q}$ obey the relationship $\hat{F}=\hat{P}\hat{Q}$. We will refer to this constraint as the *Equality* constraint. The second obvious requirement is that entries of matrices $\hat{P}$ and $\hat{Q}$ obey the probabilistic constraints of the admixture model:

$$p_{ij}\in \left[0,1\right]\;\forall \left(i,j\right)$$ (2)

$$q_{ij}\geq 0\forall \left(i,j\right)$$ (3)

$$\sum_{i}q_{ij}=1\forall j$$ (4)

As we will encounter these constraints frequently, we refer to Equation 2 as the “□” constraint, and Equation 3 and Equation 4 as the “△” constraint. This is simply because the constraints on P demarcate the boundaries of a *d*-dimensional unit cube (the generalization of a square), whereas the constraints on Q demarcate a *d*-dimensional simplex (the generalization of a triangle). Together we refer to the □ and △ constraints as the *Boundary* constraints.

The final constraint we require is that the row vectors of $\hat{F}$ lie in the linear subspace spanned by the rows vectors of Q. If we denote 〈A〉 to be the rowspace of a matrix A, we can summarize this condition as:

$$\langle \hat{F}\rangle =\langle Q\rangle$$ (5)

We will refer to Equation 5 as the *LS* (linear subspace) constraint. The LS constraint is the only nontrivial constraint that ALStructure enforces. The fact that 〈F〉=〈Q〉 is a simple consequence of the linearity of the admixture model; indeed, all rows of F are linear combinations of rows of Q since F=PQ. The LS constraint thus requires the same property for our estimate $\hat{F}$. It is important to note that the LS constraint is not the same as requiring that $\langle \hat{F}\rangle =\langle \hat{Q}\rangle$: this is ensured by the Equality constraint. Rather, the LS constraint requires that the row vectors of $\hat{F}$ belong to the rowspace of the true Q matrix. The apparent challenge of enforcing the LS constraint is that, *a priori*, one does not have access to 〈Q〉. However, ALStructure takes advantage of a recent result from Chen and Storey (2015) that 〈Q〉 can be consistently estimated directly from the data matrix X in the asymptotic regime of interest, when the number of SNPs *m* grows large. The result of Chen and Storey (2015) is, in fact, much more general than is needed in our setting, and, therefore, will likely be useful in many other problems. Because of its importance to this work, we further discuss this result in the context of the admixture model in *Latent subspace estimation*, and show that a modified PCA of X consistently recovers 〈Q〉.

### Leveraging constraints to estimate $\hat{F}$

The key step in ALStructure is to note that enforcing the LS constraint provides us with an immediate estimate for F. To motivate our estimator, first observe that the simple estimate $\tilde{F}=\frac{1}{2}X$ is, in some sense, a reasonable approximation of F: it is unbiased since $f_{ij}=\frac{1}{2}\text{E}\left[x_{ij}\right]$ under the admixture model. However, this estimate leaves much to be desired—most importantly, the estimate $\tilde{F}$ will in general be of full rank (*n*) rather than of low rank (*d*) and it will have a large variance. Assuming, for now, that we are provided with the true rowspace 〈Q〉 of F, a natural thing to try is to project the rows of $\frac{1}{2}X$ onto this linear subspace. Below, we show that this estimator has some appealing properties.

Let us denote the operator ${\text{Proj}}_{\langle S\rangle }\left(X\right)$ such that the rows of the matrix X are projected onto the linear subspace 〈S〉. (Note that the notation ${\text{Proj}}_{\langle S\rangle }\left(X\right)$ typically refers to projection of the columns of X onto the linear subspace 〈S〉, but here we use this notation to denote projection of the rows of X onto 〈S〉.) If we are given an orthonormal basis $\left\{s_{i}\right\}$ of the *d*-dimensional linear subspace 〈S〉, then:

$${\text{Proj}}_{\langle S\rangle }\left(X\right)\equiv X\left(\sum_{i=1}^{d}s_{i}s_{i}^{T}\right)$$

Lemma 1 below provides us a simple condition under which estimators of F formed by such projections are unbiased.

#### Lemma 1:

For a rank *d* matrix *F* that admits a factorization *F=PQ,* and a random matrix *X* such that *$\frac{1}{2}\text{E}\left[X\right]=F,$* any estimator of *F* of the form *${\hat{F}}_{\langle S\rangle }\equiv \frac{1}{2}{\mathrm{Proj}}_{\langle S\rangle }\left(X\right)$* is unbiased if, and only if, *〈Q〉⊆〈S〉*.

Lemma 1 is proved in Appendix A.1. In particular we note that

$$\hat{F}\equiv {\hat{F}}_{\langle Q\rangle }$$ (6)

is unbiased.

In addition to being unbiased, the estimator $\hat{F}$ is optimal in the following sense. Among all unbiased estimators constructed by projecting X onto a linear subspace, $\hat{F}$ minimizes a matrix equivalent of the mean squared error.

#### Lemma 2:

For a rank *d* matrix *F* that admits a factorization *F=PQ,* and a random matrix *X,* such that *$\frac{1}{2}\text{E}\left[X\right]=F$*, the estimator *$\hat{F}\equiv \frac{1}{2}{\mathrm{Proj}}_{\langle Q\rangle }\left(X\right)$* is an unbiased estimator of *F* and has the smallest risk among all unbiased estimators of the form *$\tilde{F}\equiv \frac{1}{2}{\mathrm{Proj}}_{\langle S\rangle }\left(X\right)$*. We define the risk to be the expectation of the squared Frobenius norm:

$$R\left(\hat{F},F\right)=\text{E}\left[\|\hat{F}-F\|{}^{2}\right]$$

$$=\text{E}\left[\sum_{ij}{\left({\hat{f}}_{ij}-f_{ij}\right)}^{2}\right]$$

Lemma 2 is proved in detail in Appendix A.2; however, the basic intuition is straightforward. Projecting X onto a linear space 〈S〉⊂〈Q〉 is biased (by Lemma 1). While projecting X onto a space 〈S〉⊃〈Q〉 will result in an unbiased estimate of F (again, by Lemma 1), dimensions orthogonal to 〈Q〉 fit noise, increasing the variance (and therefore the mean squared error) of the estimate.

We note that this strategy is related to the strategy taken in Hao *et al.* (2016), in which F was estimated by projecting $\frac{1}{2}X$ onto the space spanned by the first *d* principal components. In that work, it was observed that this simple strategy of estimating F typically outperformed existing methods. We will see in *Latent subspace estimation* that the space spanned by the first *d* principal components is a good estimator for 〈Q〉 itself, but it can be improved practically and with theoretical guarantees by performing a modified PCA. Therefore, Lemma 2 provides a theoretical justification for the empirically accurate method put forward in Hao *et al.* (2016).

### Latent subspace estimation

We have shown that the linear subspace 〈Q〉 can be leveraged to provide a desirable estimate of F. However, as 〈Q〉 is a linear subspace spanned by latent variables, it is not directly observable and must be estimated. Here, we show how a general technique developed in Chen and Storey (2015), which we will refer to as *Latent Subspace Estimation* (LSE), can be used to compute a consistent estimate of 〈Q〉 from the observed data X.

LSE is closely related to PCA, a popular technique that identifies linear combinations of variables that sequentially maximize variance explained in the data (Jolliffe 2002). As PCA is commonly used to find low-dimensional structure in high-dimensional data, a natural approach to estimating 〈Q〉 would be to employ SNP-wise PCA. More specifically, we might consider the linear space spanned by the first few eigenvectors of the n×n matrix, $\frac{1}{m}X^{T}X$ as an estimate of 〈Q〉.

The LSE-based estimate of 〈Q〉 almost exactly matches this PCA-based intuition. The only difference is that LSE accounts for the heteroscedastic nature of the admixture model, as detailed in Chen and Storey (2015). LSE has the theoretical advantage of asymptotically capturing 〈Q〉 in the high-dimensional setting (*i.e.*, as m→∞). Theorem 1, as stated here, is a special case of a more general theorem from Chen and Storey (2015), rewritten here for the special case of the admixture model.

#### Theorem 1:

Let us define *${\hat{\delta }}_{j}=\frac{1}{m}\sum_{i}2x_{ij}-x_{ij}^{2},$* and let *D* be the diagonal matrix with jth entry equal to *${\hat{\delta }}_{j}$*. The *d* eigenvectors *$\left\{\upsilon_{1},\ldots ,\upsilon_{d}\right\},$* corresponding to the top *d* eigenvalues of the matrix *$G=\frac{1}{m}X^{T}X-D,$* span the latent subspace *〈Q〉* in the sense that

$$\underset{m\to \infty }{\text{lim}}\langle \left\{v_{1},\ldots ,v_{d}\right\}\rangle △\langle Q\rangle =\emptyset$$

with probability 1, where *△* denotes the symmetric set difference. Further, the smallest *n−d* eigenvalues of *G* converge to 0 with probability 1.

Theorem 1 provides us with a simple procedure for estimating 〈Q〉 directly from data. One first computes ${\hat{\delta }}_{j}$ and constructs the n×n matrix D. Next, an eigen-decomposition of the adjusted matrix $\frac{1}{m}X^{T}X-D$ is computed. Finally, we estimate 〈Q〉 as

$$\hat{\langle Q\rangle }=\langle V_{\left(1:d\right)}^{T}\rangle$$ (7)

where $V_{1:d}$ are the first *d* columns from the singular value decomposition of G.

We stress that the general form of Theorem 1 from Chen and Storey (2015) makes LSE applicable to a vast array of models beyond factor models and the admixture model discussed here. As a further benefit to the LSE methodology, it is both easy to implement and computationally appealing. The entire computation of $\hat{\langle Q\rangle }$ requires a single eigen-decomposition of an n×n matrix, where the accuracy depends only on large *m*.

### Leveraging constraints to estimate P and Q

Now that we have a method for obtaining the estimate $\hat{F}$ by leveraging the LS constraint, what remains is to find estimates for P and Q. Since the estimate $\hat{F}$ has several appealing properties, as outlined in *Leveraging constraints to estimate $\hat{F}$*, the approach of ALStructure is simply to keep $\hat{F}$ fixed and seek matrices $\hat{P}$ and $\hat{Q}$ that obey the Equality and Boundary constraints of the admixture model. Below we discuss some of the general properties of this approach: namely the question of existence and uniqueness of solutions. We will briefly discuss the general problem of nonidentifiability in the admixture model, and provide simple and interpretable conditions under which the admixture model is identifiable. Finally, we will provide simple algorithms for computing $\hat{P}$ and $\hat{Q}$ from $\hat{F}$ based on the method of ALS.

#### *Existence*, *uniqueness*, *and anchor conditions:*

First, we develop some terminology. We will say that an m×n matrix A admits an admixture-factorization if the following feasibility problem has a solution:

find:(B,C) (8)

subject to:A=BC and(□,△)

In words, the feasibility problem in Equation 8 simply seeks a factorization of A that obeys the Equality and Boundary constraints from *Optimal model constraints* imposed by the admixture model. The smallest integer *d* for which (B,C) is a solution to Equation 8 with B an m×d matrix and C a d×n matrix is the *admixture-rank* of A, which we denote ${\text{rank}}_{\text{ADM}}\left(A\right)$. By seeking a rank *d* admixture-factorization of $\hat{F}$, ALStructure converts a problem of high-dimensional statistical inference to a matrix factorization problem.

This simple approach has two apparent shortcomings:

- A rank *d* admixture-factorization of $\hat{F}$ may not exist.
- If a valid factorization exists, it will not be unique.

Item (i) is a technical problem; though F admits a rank *d* admixture factorization by assumption, the same is not true for $\hat{F}$ in general. Even though the rank of $\hat{F}$ is *d* by construction, $\text{rank}\left(\hat{F}\right)\neq {\text{rank}}_{\text{ADM}}\left(\hat{F}\right)$ in general. ALStructure avoids this problem by changing the feasibility problem expressed in Equation 8 to the following optimization problem:

$$\underset{\left(B,C\right)}{\text{minimize}}\|A-\mathrm{BC}\|$$ (9)

subject to:(□,△)

It is important to note that (ii) is not a problem unique to ALStructure, but is a fundamental limitation for any ML method as well. This is because the likelihood function depends on $\hat{P}$ and $\hat{Q}$ only through their product $\hat{F}$; more formally, the admixture model is nonidentifiable. One unavoidable source of nonidentifiability is that any solution $\left(\hat{P},\hat{Q}\right)$ to the matrix factorization problem in Equation 8 will remain a valid solution after applying a permutation to the columns of $\hat{P}$ and the rows of $\hat{Q}$. A natural question to ask is: “When is there a unique factorization $\hat{F}=\hat{P}\hat{Q}$ up to permutations?”

**Figure 1:**
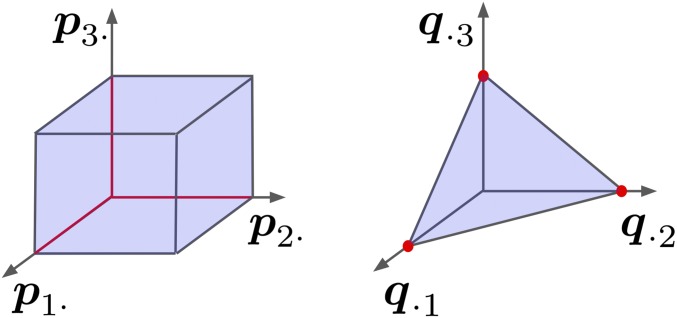
Summary of sufficient conditions for a factorization F=PQ to be unique for d=3. Axes represent the components of the row vectors of P and the column vectors of Q respectively. (left) Anchor SNPs: there is at least one row of P on each of the red lines. (right) Anchor genotypes: there is at least one column of Q on each of the red dots.

Two important types of sufficient conditions under which unique factorizations exist up to permutations are *anchor SNPs* and *anchor individuals*. We note that the concept of anchors has been previously employed in the field of topic modeling, where *anchor words* are of interest (Arora *et al.* 2013). We define an anchor SNP as one that is fixed in all ancestral populations except one. The anchor SNPs condition is then satisfied if each of the *d* ancestral populations has at least one corresponding anchor SNP. Analogously, we define an anchor individual as one whose entire genome is inherited from a single ancestral population. The anchor individuals condition is then satisfied if each of the *d* ancestral populations has at least one corresponding anchor individual. The assumption of anchor individuals is equivalent to the assumption of “surrogate ancestral samples” required by the EIGMIX method of Zheng and Weir (2016). The fact that either a set of *d* anchor SNPs or *d* anchor individuals makes the admixture model identifiable up to permutations follows from a simple argument found in Appendix A.3. For the special case of d=3, Figure 1 graphically displays the anchor conditions. It is important to remember that ALStructure does not *require* anchors to function. Rather, anchors provide interpretable conditions under which solutions provided by ALStructure, or any likelihood-based method, can be meaningfully compared to the underlying truth.

The anchor SNP and anchor individual conditions are not necessarily the only sufficient conditions for ensuring identifiability of the admixture model, and indeed, to the best or our knowledge, there is not currently a complete characterization of conditions for which the admixture model is identifiable. We regard this as an important open problem. In practice, ALStructure is capable of retrieving solutions remarkably close to the underlying truth even in simulated scenarios far from satisfying the anchor conditions, including conditions that are challenging for existing methods.

#### Computation:

Here, we present two simple algorithms for solving the optimization problem:

$$\underset{\left(P,Q\right)}{\text{minimize}}\left\|\hat{F}-\mathrm{PQ}\right\|$$ (10)

subject to:(□, △)

The first algorithm, which we call cALS (constrained ALS), has the advantage that it is guaranteed to converge to a stationary point of the nonconvex objective function in (10). While a stationary point will not generally correspond to a globally optimal solution, global optimization is seldom possible for nonconvex problems.

Although theoretically appealing, this algorithm relies on solving many constrained quadratic programming problems and is, consequently, potentially slow. To overcome this problem, we introduce a second algorithm called tALS (truncated ALS), which simply ignores the problematic quadratic constraints in cALS. Though lacking a theoretical guarantee of convergence, the increase in speed is significant and the outputs of the two algorithms are often practically indistinguishable.

We note that the general method of ALS is not novel. In particular, previous work has developed ALS methods for the problem of nonnegative matrix factorization (NNMF) (Paatero and Tapper 1994; Lee and Sebastian 1999). In NNMF, one seeks a low-rank factorization, A=BC, in which all elements of the factors B and C are non-negative. Algorithms analogous to cALS and tALS, but with non-negativity constraints rather than the □ and △ constraints, have previously been considered (Berry *et al.* 2007; Cichocki *et al.* 2007; Gillis and Glineur 2012; Kim *et al.* 2014).

#### An algorithm with provable convergence:

While problem (10) is nonconvex as stated, the following two subproblems are convex:

$$\underset{P}{\text{minimize}}\left\|\hat{F}-\mathrm{PQ}\right\|$$ (11)

subject to:□

$$\underset{Q}{\text{minimize}}\left\|\hat{F}-\mathrm{PQ}\right\|$$ (12)

subject to:△That (11) and (12) are convex problems is clear; norms are always convex functions, and □ and △ are convex constraints. In particular, (11) and (12) are both members of the well-studied class of Quadratic Programs (QP), for which many efficient algorithms exist (Boyd and Vandenberghe 2009). We propose the following procedure for factoring $\hat{F}$, which we call *Constrained ALS*.

**Algorithm 1: Constrained ALS Algorithm**

**procedure** cALS$\left(\hat{F},d\right)$.
 Initialize $\hat{P}$ arbitrarily.
 **repeat**.
  Solve (12) with $P=\hat{P}$ and return $\hat{Q}$.
  Solve (11) with $Q=\hat{Q}$ and return $\hat{P}$.
 **until** Convergence of $\hat{P}$ and $\hat{Q}$.
**return** $\left(\hat{P},\hat{Q}\right)$.

Despite the original problem being nonconvex, Algorithm 1 is guaranteed to converge to a stationary point of the objective function in (10) as a result of the following theorem from Grippo and Sciandrone (2000).

#### Theorem 2:

For the two block problem,

$$\underset{P,Q}{\mathrm{minimize}}f\left(P,Q\right)$$

if *$\left\{P_{i}\right\}$* and *$\left\{Q_{i}\right\}$* are sequences of optimal solutions to the two subproblems:

$$\underset{P}{\mathrm{minimize}}f\left(P,Q_{i}\right)$$

$$\underset{Q}{\mathrm{minimize}}f\left(P_{i},Q\right)$$

then any limit point *(P,Q)* will be a stationary point of the original problem. (Note that the result from Grippo and Sciandrone (2000) is actually more general than this. We reproduce the special case above in order to make clear the connection to our problem.)

#### An efficient heuristic algorithm:

If we remove all constraints on P and Q from Equation 11 and Equation 12, the resulting optimization problems are simple linear LS.

$$\underset{P}{\text{minimize}}\left\|F-\mathrm{PQ}\right\|$$ (13)

$$\underset{Q}{\text{minimize}}\left\|F-\mathrm{PQ}\right\|$$ (14)

Our algorithm proceeds by alternating between solving the unconstrained LS problems (13) and (14). After each step, the optimal solution will not necessarily obey the constraints of problem (10). To keep our algorithm from converging on an infeasible point, we truncate the solution to force it into the feasible set. More precisely, each element of the solution $P^{*}$ to (13) is truncated to satisfy □ and each column of the solution $Q^{*}$ to (14) is projected to the closest point on the simplex defined by the △ constraint. Simplex-projection is nontrivial; however, it is a well-studied optimization problem. Here we use a particularly simple and fast algorithm from Chen and Ye (2011). This algorithm, which we call the *Truncated ALS Algorithm*, is detailed in Algorithm 2.

**Algorithm 2: Truncated ALS Algorithm**

**procedure** tALS$\left(\hat{F},d\right)$
 Initialize $\hat{P}$ arbitrarily.
 **repeat**
  Solve (14) with $P=\hat{P}$, and return the simplex-projected solution $\hat{Q}$.
  Solve (13) with $Q=\hat{Q}$ and return the truncated solution $\hat{P}$.
 **until** Convergence of $\hat{P}$ and $\hat{Q}$
**return** $\left(\hat{P},\hat{Q}\right)$

#### An example dataset:

**Figure 2:**
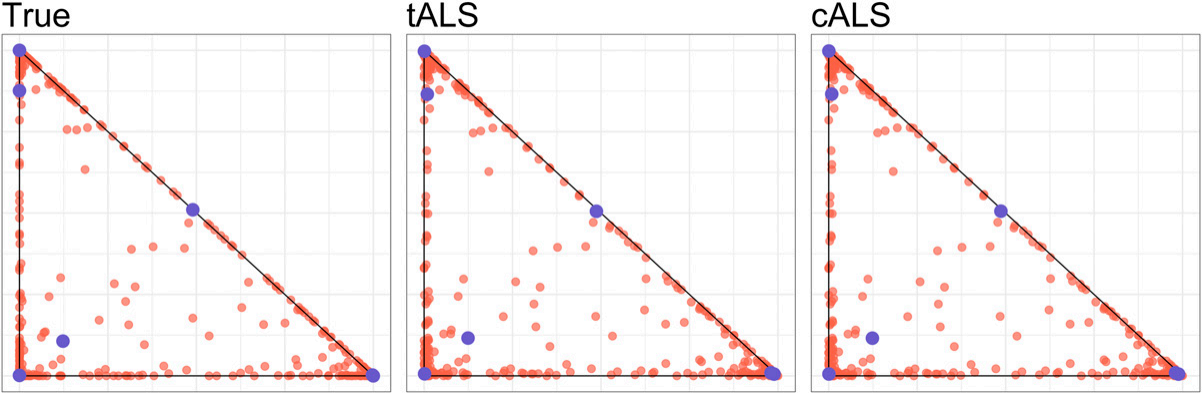
Biplots of the first two rows of Q (left), ${\hat{Q}}_{\text{tALS}}$ (middle) and ${\hat{Q}}_{\text{cALS}}$ (right). Blue points are provided as a visual aid and delineate a common subset of individuals.

Figure 2 displays the output of cALS and tALS on a dataset from the PSD model with the parameters: m=100,000, n=500, d=3, α=(0.1,0.1,0.1). As can be seen, the output fits for Q provided by cALS and tALS are practically indistinguishable to the eye, and are both excellent approximations of the ground truth. The cALS algorithm performed slightly better than the tALS algorithm ($8.5\times 10^{-3}$ and $8.7\times 10^{-3}$ RMSE, respectively). However, cALS took 3.5 hr to complete while tALS terminated in under 1.5 min. Because of the significant gains in efficiency, we use tALS exclusively throughout the remainder of this paper. The analyst who requires theoretical guarantees can, of course, use the cALS algorithm instead. Appendix B provides a more detailed comparison between the tALS and cALS algorithms on simulated data.

## The ALStructure algorithm

In this section, we briefly outline the entire ALStructure method whose components were motivated in depth in *Model and Theory*. In order to fit the admixture model, we obtain estimates $\hat{F}$, $\hat{P}$, and $\hat{Q}$ from the SNP matrix X through the following three-part procedure:

- Estimate the linear subspace 〈Q〉 from the data X.
- Project $\frac{1}{2}X$ onto the estimate $\hat{\langle Q\rangle }$ to obtain an estimate of F.
- Factor the estimate $\hat{F}$ subject to the Equality and Boundary constraints to obtain $\hat{P}$ and $\hat{Q}$.

For convenience, we detail the entire ALStructure algorithm in Algorithm 3, and annotate each of the three steps described above. We note that we have decided to use the tALS function rather than the cALS function in our definition of the ALStructure algorithm, valuing the speed advantage of tALS over the theoretical guarantees of cALS. If desired, one could of course choose to use the cALS function instead without making any other alterations to the ALStructure.

**Algorithm 3: ALStructure**

**procedure** ALStructure(X,d)
 **for** j=0 to n do                (i)
  ${\hat{\delta }}_{j}\leftarrow \frac{1}{m}\sum_{i=1}^{m}2x_{ij}-x_{ij}^{2}$
 $D\leftarrow \text{diag}\left(\left\{{\hat{\delta }}_{1},\ldots ,{\hat{\delta }}_{n}\right\}\right)$
 $G\leftarrow \frac{1}{m}X^{T}X-D$
 Compute eigen-decomposition $G=\mathrm{VW}V^{T}$
 $\hat{F}\leftarrow \frac{1}{2}{\text{Proj}}_{\hat{\langle Q\rangle }}\left(X\right)=\frac{1}{2}XV_{\left(1:d\right)}V_{\left(1:d\right)}^{T}V_{\left(1:d\right)}^{T}$       (ii)
 $(\hat{P},\hat{Q})\leftarrow \text{tALS}\left(\hat{F},d\right)$               (iii)
**return** $\left(\hat{F},\hat{P},\hat{Q}\right)$

We emphasize here that ALStructure’s estimate $\hat{Q}$ is ultimately derived from the LSE-based estimate of the latent subspace $\hat{\langle Q\rangle }$. As the method of LSE is closely linked to PCA, we consider ALStructure to be a unification of PCA-based and likelihood-based approaches.

Perhaps the most striking feature of Algorithm 3 is its brevity. One advantage of this simplicity is its ease of implementation. Although Algorithm 3 has been implemented in the R package ALStructure, it can clearly be reimplemented in any language quite easily. Equally important is that all of the operations in Algorithm 3 are standard. The only two computationally expensive components are a single eigen-decomposition if *n* is large, and QR decompositions to find linear least squares (LLS) solutions in the tALS algorithm. Both of these problems have a rich history and consequently have many efficient algorithms. It is likely that the ALStructure implementation of Algorithm 3 can be significantly sped up by utilizing approximate or randomized algorithms for the eigen-decomposition and/or LLS computations. In its current form, ALStructure simply uses the base R functions eigen() and solve() for the eigen-decomposition and LLS computations, respectively. Despite this, the current implementation of ALStructure is typically faster than existing algorithms as can be seen in *Results From Simulated Data* and *Applications to Global Human Studies* below.

The ALStructure method is a nonparametric estimator in the following ways. It makes no assumptions about the probability distributions of P or Q. Any random variable taking values in {0,1} is by necessity Bernoulli. In this vein, the assumption that $x_{ij}∼\text{Binomial}\left(2,f_{ij}\right)$ is not a parametric assumption *per se*, but rather an assumption about independence of alleles. Finally, the likelihood function is not utilized in estimating P and Q, making ALStructure likelihood-free.

For choosing the dimensionality of the model *d*, we recommend utilizing the recently proposed structural Hardy-Weinberg equilibrium (sHWE) test (Hao and Storey 2017). This test can perform a genome-wide goodness of fit test to the assumptions made in the admixture model over a range of *d*. It then identifies the minimal value of *d* that obtains the optimal goodness of fit. There are other ways to choose *d*, by using the theory and methods in Chen and Storey (2015) or by using other recent proposals (Patterson *et al.* 2006; Hao *et al.* 2016).

## Results from Simulated Data

### Simulated data sets

In this section, we compare the performance of ALStructure to three existing methods for global ancestry estimation, Admixture, fastSTRUCTURE and terastructure. Admixture, developed by Alexander *et al.* (2009), is a popular algorithm that takes a ML approach to fit the admixture model. Both fastSTRUCTURE (Raj *et al.* 2014) and terastructure (Gopalan *et al.* 2016) are Bayesian methods that fit the PSD model using variational Bayes approaches. We abbreviate these methods as ADX, FS, and TS in the figures. A comparison among these three methods appears in Gopalan *et al.* (2016), so we will focus on how they compare to ALStructure.

**Figure 3:**
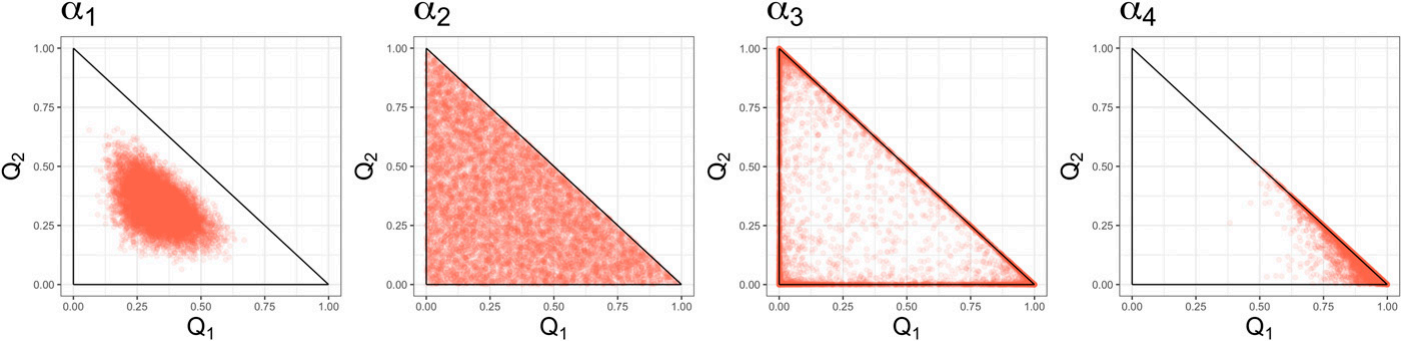
Examples of typical random samples from the four different α-prototypes. As can be seen, only $\alpha_{2}$ and $\alpha_{3}$ approximately obey the “anchor-individuals” condition.

To this end, we first tested all algorithms on a diverse array of simulated datasets. The bulk of our simulated data sets come from the classical PSD model (defined in *The admixture model*), in which columns of Q are distributed according to the Dirichlet(α) distribution and the rows of P are drawn from the Balding-Nichols distribution. We varied the following parameters in our simulated datasets: *m*, *n*, *d*, and α. Of particular note is the variation of α. For this we used four α-prototypes: $\alpha_{1}=\left(10,10,10\right)$, $\alpha_{2}=\left(1,1,1\right)$, $\alpha_{3}=\left(0.1,0.1,0.1\right)$, and $\alpha_{4}=\left(10,1,0.1\right)$. These four prototypes were chosen because they represent four qualitatively different distributions on the Dirichlet simplex as shown in Figure 3: $\alpha_{1}$ corresponds to points distributed near the center of the simplex, $\alpha_{2}$ corresponds to points distributed evenly across the simplex, $\alpha_{3}$ corresponds to points distributed along the edges of the simplex, and $\alpha_{4}$ corresponds to an asymmetric distribution in which points are concentrated in one of the corners of the simplex.

When we produced datasets with d>3, we extended the prototypes in the natural way; for example for d=6, the $\alpha_{4}$ is becomes (10,10,1,1,0.1,0.1). Table 1 lists all of the parameters we used to generate data under the Dirichlet model, for a total of 96 distinct combinations.

**Table 1:** Parameters of all simulated datasets

Parameters	
*m*	$10^{5}$, $5\times 10^{5}$
*n*	$5\times 10^{2}$, $10^{3}$, $5\times 10^{3}$, $10^{4}$
*d*	3, 6, 9
α-prototypes	(10, 10, 10)
	(1, 1, 1)
	(0.1, 0.1, 0.1)
	(10, 1, 0.1)

The parameters of the Balding-Nichols distributions from which rows of the P matrix were drawn were taken from real data, following the same strategy taken in Gopalan *et al.* (2016). Specifically, $F_{i}$ and $p_{i}$ were estimated for each SNP in the Human Genome Diversity Project (HGDP) dataset (Cavalli-Sforza 2005). Then, for each simulated dataset, *m* random samples are taken (with replacement) from the HGDP parameter estimates.

In addition to simulating Q matrices from the classical Dirichlet(α) distribution with many different parameters α, we also simulated data from the spatial model of admixture developed in Ochoa and Storey (2016). We deliberately chose to study this model because it is ill-suited for ALStructure; while ALStructure relies on the estimation of the *d*-dimensional linear subspace 〈Q〉, the columns of Q produced under the spatial model lie on a one-dimensional curve within 〈Q〉. Despite this fundamentally challenging scenario, we see that ALStructure is often capable of recovering an accurate approximation.

### Results from the PSD model

In order to give a representative picture of the relative performance of ALStructure against existing algorithms, we first plot the fits of all of the algorithms for two particular data sets out of the total 96 model data sets: (i) the data set in which ALStructure performs the best and (ii) the data set in which ALStructure performs the worst, according to mean absolute error (defined below).

**Figure 4:**
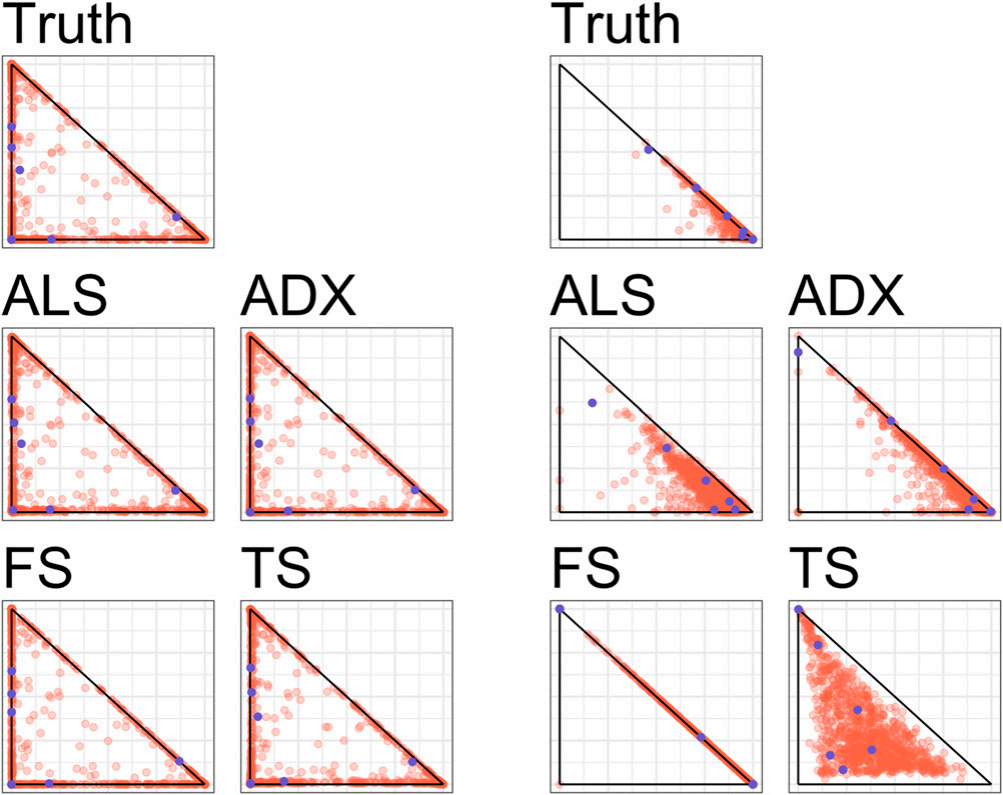
Model fits by ALStructure, Admixture, fastSTRUCTURE, and terastructure on two particular simulated datasets. The left panel shows the fits corresponding to the dataset on which ALStructure performed the best. The right panel shows the fits corresponding to the dataset on which ALStructure performed the worst. Each point represents a column of the Q matrix and is plotted by the first and second coordinates. Blue points are plotted as a visual aid and delineate a common subset of individuals.

On the left side of Figure 4, we see that all four algorithms perform very well for the data set in which ALStructure performs best, which comes from the $\alpha_{3}$-prototype. On the right side of Figure 4, the dataset was generated from the $\alpha_{4}$-prototype. We see that, while ALStructure certainly deviates substantially from the truth, so does every algorithm. Both fastSTRUCTURE and terastructure provide results that are qualitatively very different from the truth; where fastSTRUCTURE compresses all columns of Q onto a single edge of the simplex, terastructure spreads them out through the interior of the simplex. Both Admixture and ALStructure provide solutions qualitatively similar to the truth. While the points in the Admixture solution extend much further along the edge of the simplex than the true model, the ALStructure solution spreads into the interior of the simplex more than the true model.

**Figure 5:**
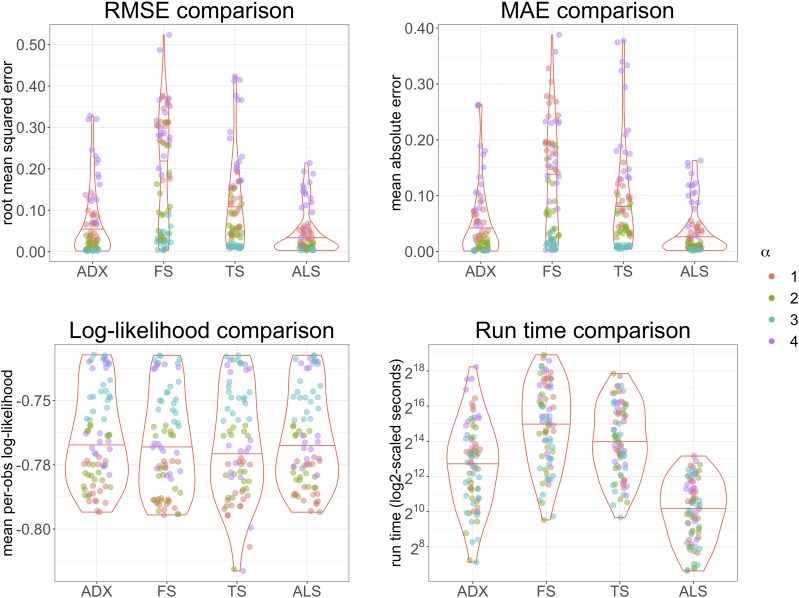
Summary of performance of ALStructure and existing algorithms. The points are colored by α-prototype.

Figure 5 provides a comprehensive summary of the performance of ALStructure against the existing algorithms on all simulated datasets. The top panels of Figure 5 summarize the accuracy of each of the four algorithms, according to two metrics: root mean squared error (RMSE) and mean absolute error (MAE).

$$\text{RMSE}\equiv \sqrt{\frac{1}{dn}\sum_{k=1}^{d}\sum_{j=1}^{n}{\left({\hat{q}}_{kj}-q_{kj}\right)}^{2}}$$

$$\text{MAE}\equiv \frac{1}{dn}\sum_{k=1}^{d}\sum_{j=1}^{n}\left|{\hat{q}}_{kj}-q_{kj}\right|$$

The bottom left panel of Figure 5 shows mean per observation log-likelihood, $\frac{1}{mn}\sum_{i=1}^{m}\sum_{j=1}^{n}\text{log }P\left(x_{ij}{|\hat{f}}_{ij}\right)$, on all simulated data sets. (To obtain full data log-likelihoods, multiple these numbers by mn.) It is interesting to note that ALStructure performs comparably to other methods from the likelihood perspective despite the fact that it is the only method that does not explicitly utilize the likelihood function. However, we emphasize that likelihood is an imperfect metric of model fit for two reasons. First, because of the highly nonidentifiable nature of the admixture model as discussed in *Model and Theory*, many models are equivalent from the likelihood perspective. Therefore, if one is primarily concerned about the accuracy of admixture estimates, the RMSE or MAE metrics may be more suitable. Second, in high-dimensional models, it has been demonstrated that high likelihood may yield far inferior estimates (Efron 2013). Starting with Stein’s Paradox (Stein 1956), it has been shown in many settings that the ML estimator for several parameters may be uniformly worse in accuracy than methods that leverage shared information in the data.

The bottom right panel of Figure 5 shows the distributions of run times for each algorithm on all modeled datasets. Due to the size of the simulated datasets and our computational constraints, each algorithm did not terminate on each of the 96 datasets. In Figure 5, we plot only the datasets for which all four algorithms successfully terminated. See Appendix C for more details. It is clear that ALStructure is competitive with respect to both model fit and time. ALStructure outperforms all methods according to both RMSE and MAE. With respect to time, ALStructure is clearly favored (one should note that the *y*-axis is on the log scale).

### Results from the spatial model

As a challenge to ALStructure, we simulate data from a model developed in Ochoa and Storey (2016), which we will refer to as the *spatial model*. This model mimics an admixed population that was generated by a process of diffusion in a one-dimensional environment. There are *d* unmixed ancestral populations equally spaced at positions $\left\{x_{0},x_{0}+1,\ldots ,x_{0}+d-1\right\}$ on an infinite line. If all populations begin to diffuse at time t=0 at the same diffusive rate, then population *i* will be distributed as a Gaussian with mean $\mu_{i}=x_{0}+i-1$ and SD σ. Therefore, under the spatial model, an individual sampled from position *x* will have admixture proportions shown in Equation 15,

$$\left(q_{1}\left(x\right),q_{2}\left(x\right),\ldots ,q_{d}\left(x\right)\right)=\left(\frac{f_{\left(\mu_{1},\sigma \right)}\left(x\right)}{\sum_{i=1}^{d}f_{\left(\mu_{i},\sigma \right)}\left(x\right)},\frac{f_{\left(\mu_{2},\sigma \right)}\left(x\right)}{\sum_{i=1}^{d}f_{\left(\mu_{i},\sigma \right)}\left(x\right)},\ldots ,\frac{f_{\left(\mu_{d},\sigma \right)}\left(x\right)}{\sum_{i=1}^{d}f_{\left(\mu_{i},\sigma \right)}\left(x\right)}\right)$$ (15)

where $f_{\left(\mu ,\sigma \right)}$ denotes the Gaussian distribution with parameters (μ,σ).

**Figure 6:**
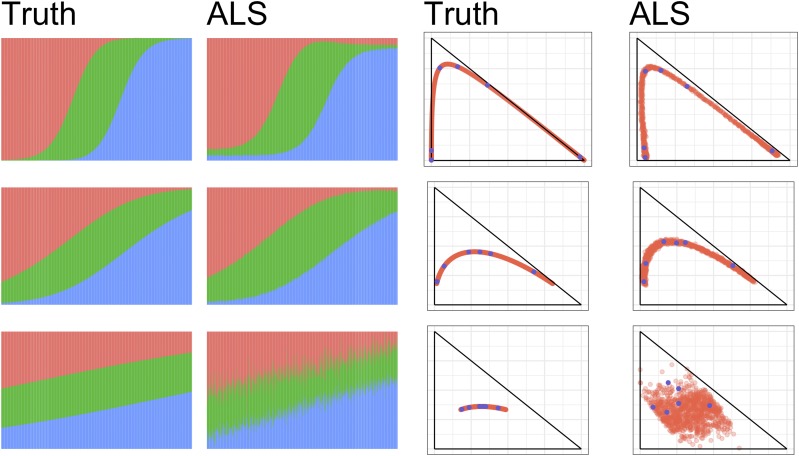
ALStructure fits of datasets from the Spatial model. (left) Stacked barplots of ALStructure fits. (right) Biplots of ALStructure fits. The parameter σ was set to 0.5, 1, and 2 for the top, middle and bottom rows, respectively. Blue points are plotted as a visual aid and delineate corresponding columns of Q and $\hat{Q}$.

Although this is just a special case of the admixture model, one would expect the spatial model to be particularly challenging for ALStructure because the admixture proportions belong to a one-dimensional curve parameterized by *x*, and ALStructure necessitates the estimation of a *d*-dimensional linear subspace in ${\mathbb{R}}^{n}$. The challenge is much more pronounced when the populations are highly admixed (large σ). Figure 6 shows the model fits provided by ALStructure. 

Indeed, for large values of σ (σ=2), ALStructure fails to correctly capture the admixture proportions. However, for smaller values of σ (σ={1,0.5}), it can be seen that the fits provided by ALStructure are excellent. In all simulations $m=10^{5}$, $n=10^{3}$, and d=3.

We note that Gopalan *et al.* (2016) tested Admixture, fastSTRUCTURE, and terastructure on data drawn from the spatial model (which they refer to as “Scenario B”). They showed this model posed a significant challenge for all three methods, but found that terastructure performed the best.

## Applications to Global Human Studies

Here, we apply ALStructure and existing methods to three globally sampled human genotype datasets: the Thousand Genomes Project (TGP), HGDP, and Human Origins (HO) datasets (Cavalli-Sforza 2005; Lazaridis *et al.* 2014; The 1000 Genomes Project Consortium *et al.* 2015). Table 2 summarizes several basic parameters of each of the datasets and Appendix D details the procedures used for building each dataset. Although we recommend using sHWE from Hao and Storey (2017) for choosing *d*, here we take the number of ancestral populations *d* directly from Gopalan *et al.* (2016) so that our results are easily comparable to those of the latter study.

**Table 2:** Dataset parameters

Dataset	*m*	*n*	*d*	m×n
TGP	1,229,310	1815	8	$∼2.2\times 10^{9}$
HGDP	550,303	940	10	$∼5.2\times 10^{8}$
HO	372,446	2251	14	$∼8.4\times 10^{8}$

**Figure 7:**
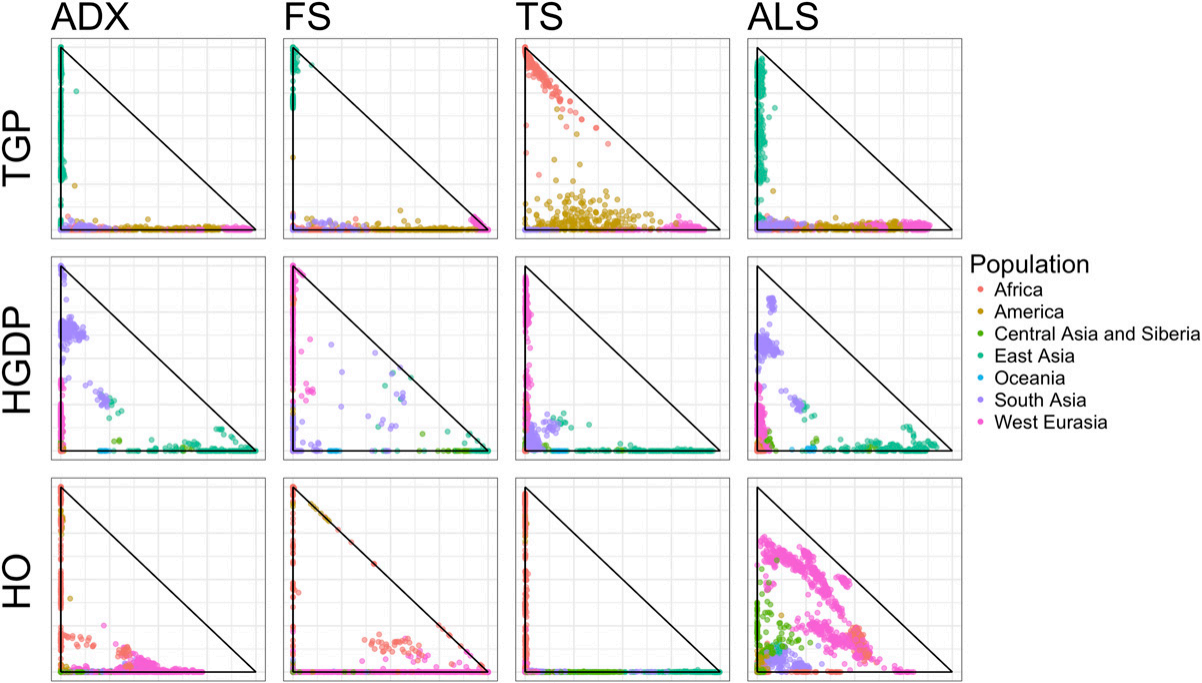
Biplots of the first two rows of Q (ranked by variation explained) of the fits of the TGP (top), HGDP (middle), and HO (bottom) datasets for each algorithm. Individuals are colored by coarse subpopulation from which they are sampled.

Figure 7 shows scatterplots of the first two rows of $\hat{Q}$ for each of the three datasets provided by each of the four fits. To disambiguate the inherent nonidentifiability (see *Model and Theory*), we ordered the rows of the fits $\hat{Q}$ by decreasing variation explained: $s_{i}^{2}={\left\|X{\hat{q}}_{i\cdot }^{T}\right\|}^{2}$. Perhaps the most striking aspect of Figure 7 is the difference between the fits produced by each method. With the notable exception that Admixture and ALStructure have similar fits for the TGP and HGDP datasets, every pair of comparable scatterplots (*i.e.*, within a single row of Figure 7) are qualitatively different. Figure 11 in the Appendix displays the same data represented as stacked barplots of the admixture proportions. In this representation too, qualitative differences between the fits are also evident. Table 3 shows the mean per observation log-likelihood of the fits provided by each of the four methods. Figure 12 in the Appendix shows that the distributions of per observation likelihood are nearly indistinguishable across all methods.

**Table 3:** The mean per-observation log-likelihood of each dataset under each method’s fit

Method	Admixture	fastSTRUCTURE	terastructure	ALStructure
TGP	−0.7097	−0.7136	−0.7130	−0.7100
HGDP	−0.7494	−0.7536	−0.7608	−0.7505
HO	−0.7467	−0.7515	−0.7534	−0.7477

Next we compare the performance of ALStructure to existing methods both in terms of efficiency and accuracy. Unlike in the case of simulated datasets where the ground truth is known, here we cannot directly compare the quality of model fits across methods. Instead, we assess the quality of each method by its performance on data simulated from real data fits.

For concreteness, we briefly outline the process below:

- Fit each dataset with each of the four methods to obtain 12 model fits.
- Simulate datasets from the admixture model using parameters obtained in the previous step.
- Fit each of the 12 simulated datasets with each of the four datasets (48 fits) and compute error measures.

The process above treats each of the four methods symmetrically, evaluating each method based on its ability to fit data simulated from both its own model fits as well as every other methods’ model fits.

**Figure 8:**
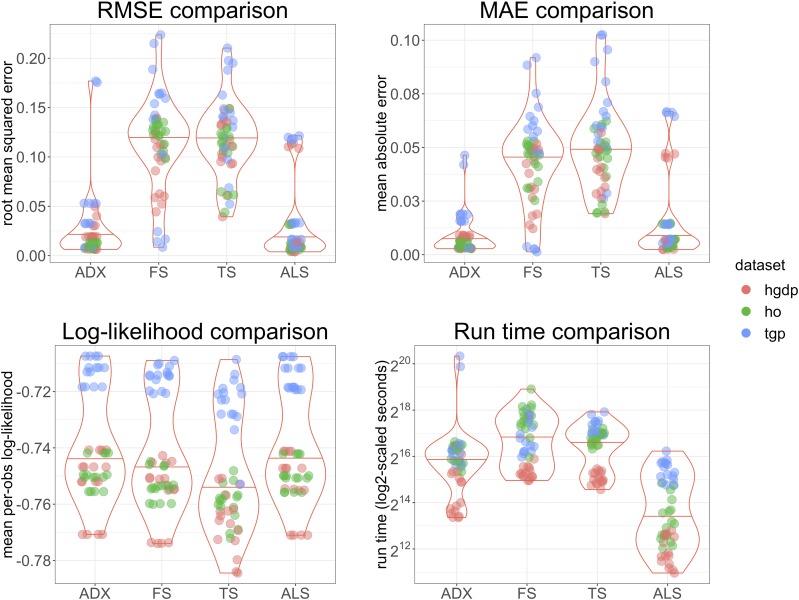
Summary of performance of ALStructure and preexisting algorithms on data simulated from real model fits. The points are colored by dataset.

Figure 8 summarizes the performance of each method with respect to both model fit and efficiency on data simulated from the above described process. As with the results on simulated datasets from *Results from the PSD model*, it is clear that ALStructure is competitive with respect to both model fit and time. Both Admixture and ALStructure outperform fastSTRUCTURE and terastructure by all quality of fit metrics. ALStructure far outperforms all methods with respect to time (one should note that the *y*-axis is on the log scale).

**Figure 10:**
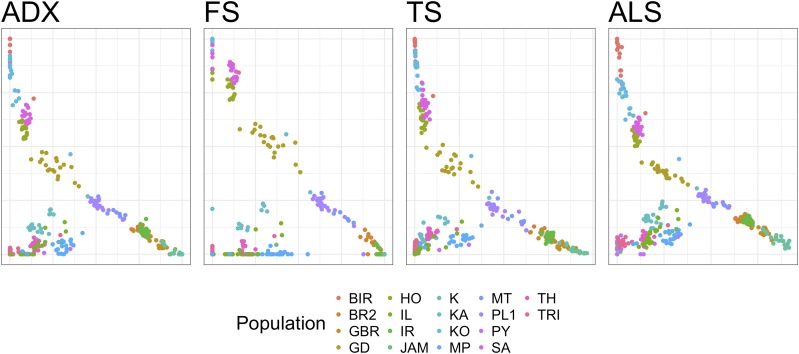
Bi-plots of the first two rows of Q (ranked by variation explained) of the fits of the Basu *et al.* (2016) dataset for each algorithm. Individuals are colored by the subpopulation from which they are sampled.

**Figure 11:**
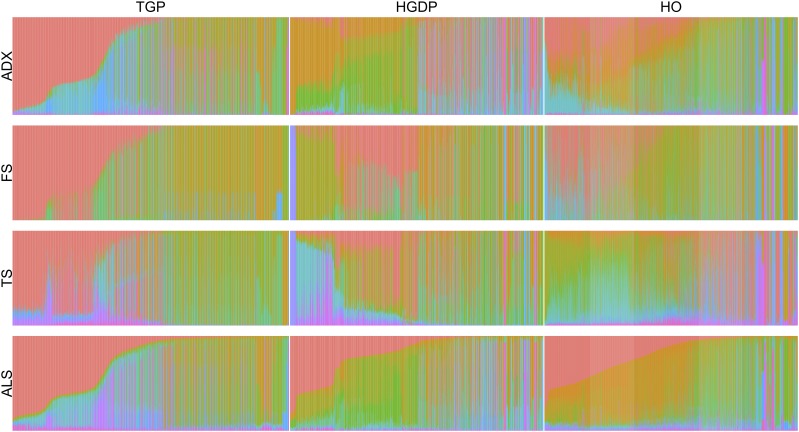
The admixture proportions of each globally sampled dataset as a stacked barplot. Because of the nonidentifiability of the model, the order of the rows of Q are arbitrary. To disambiguate this, we order the rows of Q in each dataset by decreasing average admixture. The coloring in Figure 11 is then done according to this ordering. As an aid to the eye, we also reorder the columns of Q according to decreasing proportion of the first row of Q of the ALStructure fit. The choice to order all fits according to ALStructure is arbitrary; however, all fits must be ordered consistently to make meaningful comparisons possible. As can be seen, each of the fits differ significantly from each other on every dataset.

**Figure 12:**
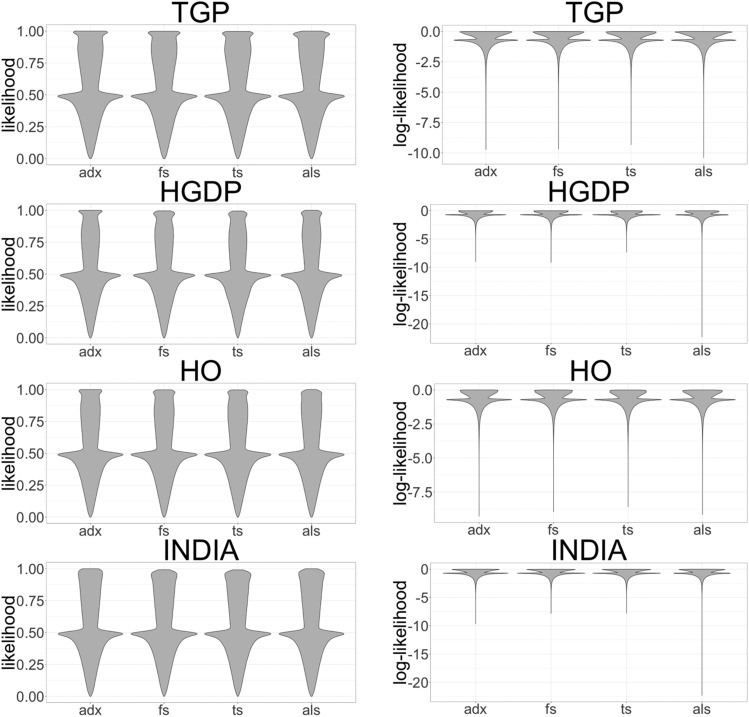
Left panels: The distribution of likelihoods for each element of X for each method and dataset. Right panels: The same as the left panels, except on a log-scale.

In Appendix E, we compare the performance of ALStructure to pre-existing methods on an additional nonglobal dataset from Basu *et al.* (2016). In this dataset, individuals are sampled from 18 modern Indian subpopulations. India’s genetic admixture is of particular interest because of its long history of sociocultural norms promoting endogamy. We find that each of the four methods produce admixture estimates qualitatively similar to each other for this dataset (see Figure 10 in the Appendix). One possible explanation for this observed similarity is that the genetic history of India more closely mimics the admixture model than does global genetic history, as suggested by Lawson *et al.* (2018).

## Discussion

In this work, we introduced ALStructure, a new method to fit the admixture model from observed genotypes. Our method attempts to find common ground between two previously distinct approaches to understanding genetic variation: likelihood-based approaches and PCA-based approaches. ALStructure features important merits from both. Like the likelihood-based approaches, ALStructure is grounded in the probabilistic admixture model, and provides full estimates of global ancestry. However, operationally the ALStructure method closely resembles PCA-based approaches. In particular, ALStructure’s estimates of global ancestry are derived from a consistent PCA-derived estimate that captures the underlying low-dimensional latent subspace. In this way, ALStructure can be considered a unification of likelihood-based and PCA-based methods.

Because ALStructure is operationally similar to PCA-based methods, it is computationally efficient. Specifically, the only computationally expensive operations required by the ALStructure algorithm are singular value and QR decompositions. Both of these computations have been extensively studied and optimized. Although ALStructure already performs favorably compared to preexisting algorithms in computational efficiency, it is likely that, by applying more sophisticated matrix decomposition techniques, ALStructure may see significant improvements in speed. Although extremely simple, ALStructure typically outperforms preexisting algorithms both in terms of accuracy and time. This observation holds under a wide array of datasets, both simulated and real.

The usefulness of PCA-based approaches has been increasingly recognized in related settings, such as the mixed membership stochastic block model (Rubin-Delanchy *et al.* 2017) and topic models (Ke and Wang 2017). The basic approach we have presented is quite general. In particular, the set of models that satisfy the underlying assumptions of LSE is large, subsuming the admixture model as well as many other probabilistic models with low intrinsic dimensionality. Consequently, we expect that the ALStructure method can be trivially altered to apply to many similar problems beyond the estimation of global ancestry.

## Figures and Tables

**Figure 9:**
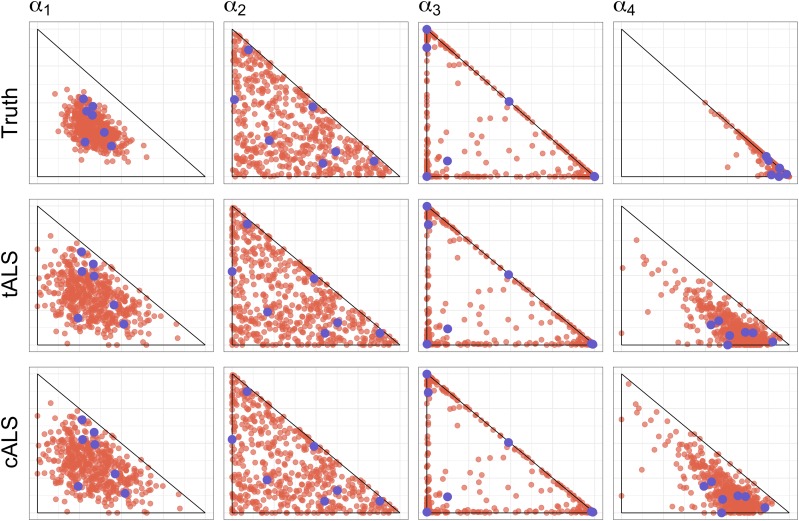
Biplots of the first two rows of Q (top), ${\hat{Q}}_{\text{tALS}}$ (middle) and ${\hat{Q}}_{\text{cALS}}$ (bottom) for each of the four α-prototypes.

## Appendix A: Additional Mathematical Details

### A.1 Proof of Lemma 1

First we show that $\hat{F}$ is unbiased. Note that:

$$\text{E}\left[\hat{F}\right]=\frac{1}{2}\text{E}[{\text{Proj}}_{\langle Q\rangle }\left(X\right)]$$

$$=\frac{1}{2}{\text{ Proj}}_{\langle Q\rangle }\left(\text{E}\left[X\right]\right)$$

$$={\text{Proj}}_{\langle Q\rangle }\left(F\right)$$

=F

Between the first and second line, we note that the projection operator is linear and take advantage of linearity of expectation. Between the second and third line, we used the observation that $\frac{1}{2}\text{E}\left[X\right]=F$. Finally, ${\text{Proj}}_{\langle Q\rangle }F=F$ since all rows of F belong to 〈Q〉. From an identical argument one can see that for projection onto any other subspace 〈S〉, the corresponding estimator ${\hat{F}}_{\langle S\rangle }\equiv \frac{1}{2}{\text{Proj}}_{\langle S\rangle }\left(X\right)$ will have the property that

$$\text{E}\left[{\hat{F}}_{\langle S\rangle }\right]={\text{Proj}}_{\langle S\rangle }\left(F\right)$$

It is clear that, if 〈Q〉⊆〈S〉, then $\text{E}{\left[\hat{F}\right]}_{\langle S\rangle }]={\text{Proj}}_{\langle S\rangle }\left(F\right)=F,$ since the projection operator acts as the identity operator for vectors belonging to the subspace 〈S〉.

Next we show that the converse is true: $\text{E}\left[{\text{Proj}}_{\langle S\rangle }\right]=F$ implies 〈Q〉⊆〈S〉. To do this, we prove the contrapositive statement. If 〈Q〉⊈〈S〉, then $\text{E}\left[{\hat{F}}_{S}\right]\neq F$. This can be seen by noting that each row in $\hat{F}={\text{Proj}}_{\langle Q\rangle }\left(F\right)$ is a vector in the linear subspace 〈Q〉 projected into the linear subspace 〈S〉; rows of $\hat{F}$ therefore belong to the linear subspace 〈Q〉∩〈S〉. Unless 〈Q〉⊆〈S〉, then the dimension of 〈Q〉∩〈S〉 is strictly less than *d*, the dimension of 〈Q〉 and the rank of F. Therefore, if 〈Q〉⊈〈S〉, the rank of $\text{E}\left[{\hat{F}}_{S}\right]$ will be less than the rank of F, implying $\text{E}\left[{\hat{F}}_{S}\right]\neq F$.

### A.2 Proof of Lemma 2

Note that we can write the squared Frobenius norm as follows:

$$L\left(F,\hat{F}\right)\equiv {\left\|\hat{F}-F\right\|}^{2}$$

$$=\text{Tr}\left[{\left(\hat{F}-F\right)}^{T}\left(\hat{F}-F\right)\right]$$

$$=\text{Tr}\left[{\hat{F}}^{T}\hat{F}\right]-2\text{Tr}\left[{\hat{F}}^{T}F\right]+\text{Tr}\left[F^{T}F\right]$$ (16)

First, let us compute the risk of our projection estimator $\hat{F}$. Suppose we have an orthonormal basis $\left\{v_{i}\right\}$ of 〈Q〉. Using the definition of $\hat{F}$ from Equation 6, and the fact that the rows of both F belong to 〈Q〉, we note that we can write any row of either matrix in terms of the basis vectors $\left\{v_{i}\right\}$:

$$f_{i\cdot }=\sum_{j}\langle f_{i\cdot }^{T},v_{j}\rangle v_{j}^{T}$$ (17)

$${\hat{f}}_{i\cdot }=\frac{1}{2}\sum_{j}\langle x_{i\cdot }^{T},v_{j}\rangle v_{j}^{T}$$ (18)

By rewriting the matrices $\hat{F}$ and F with respect to the basis $\left\{v_{i}\right\},$ and using Equation 17 and Equation 18, it is a straightforward calculation to show that

$$\text{Tr}\left[F^{T}F\right]=\sum_{i=1}^{m}\sum_{j=1}^{k}{\langle f_{i\cdot }^{T},v_{j}\rangle }^{2}$$

$$\text{Tr}\left[{\hat{F}}^{T}\hat{F}\right]=\sum_{i=1}^{m}\sum_{j=1}^{k}{\langle \frac{1}{2}x_{i\cdot }^{T},v_{j}\rangle }^{2}$$

Substituting this result into Equation 16 and taking expectations, we have the following expression for our loss function:

$$R\left(F,\hat{F}\right)=\text{E}\left[L\left(F,\hat{F}\right)\right]$$

$$=\text{E}\left[\text{Tr}\left[{\hat{F}}^{T}\hat{F}\right]-2\text{Tr}\left[{\hat{F}}^{T}F\right]+\text{Tr}\left[F^{T}F\right]\right]$$

$$=\text{E}\left[\text{Tr}\left[{\hat{F}}^{T}\hat{F}\right]-\text{Tr}\left[F^{T}F\right]\right]$$

$$=\sum_{i=1}^{m}\sum_{j=1}^{k}\text{E}\left[{\langle \frac{1}{2}x_{i\cdot }^{T},v_{j}\rangle }^{2}\right]-{\langle f_{i\cdot }^{T},v_{j}\rangle }^{2}$$

$$=\frac{1}{4}\sum_{i=1}^{m}\sum_{j=1}^{k}\text{Var}\left[\langle x_{i\cdot }^{T},v_{j}\rangle \right]$$ (19)

By studying Equation 19, we can see the estimator $\hat{F}$ has several favorable properties. First note that the risk is a sum of m×k nonnegative numbers since Var[Z]≥0 for any random variable Z. If we were to project onto a larger subspace 〈*S*〉, where 〈S〉⊃〈Q〉, we would add terms to Equation 19 and consequently increase our risk. If we were to project onto a smaller subspace 〈S〉⊂〈Q〉, then the risk may decrease; however, our new estimator will now be biased by Lemma 1. From these observations, we conclude that $\hat{F}$ is optimal in the sense described in the Lemma 2.

### A.3 Proof of sufficiency of anchors

Here, we show that either a set of anchor SNPs or a set of anchor individuals is sufficient to specify a unique factorization F=PQ up to the nonidentifiability associated with row permutations.

#### Proposition 1.

*For a rank d matrix F with a factorization F=PQ*, *if there is a set S of d rows of P such that for each i∈{1,2,…,d} there exists a row vector $p_{i\cdot }\in S$ such that $p_{i\cdot }=\delta_{i}e_{i}$ for $\delta_{i}\neq 0$*, where $e_{i}$ is a vector of length *n* in which element *i* is 1, and all other elements are 0, *then the factorization is unique up to permutation*. *When such a set S exists, we say that we have “anchor SNPs.”*

#### Proof.

Let us denote the matrix $D=\text{diag}\left(\delta_{1},\delta_{2},\ldots ,\delta_{d}\right)$. Without loss of generality, let us assume that *S* is the first *d* rows of *P* and are ordered such that

$$P=\left(\begin{array}{c} D\\ A \end{array}\right)$$

for some (m−d)×d matrix A. Then there is a unique Q for this F matrix (up to permutation) which is

$$Q=D^{-1}F_{1:d}$$

The matrix A is also uniquely determined by F once Q is fixed. To see this, note that

$$f_{j\cdot }=p_{j\cdot }Q$$

where $f_{j\cdot }$ and $p_{j\cdot }$ denote the *j* row of F and P respectively. Since $f_{j\cdot }$ is fixed and Q is unique under the anchor SNP assumption, there is a unique solution for $p_{j\cdot }$ by the linear independence of the rows of Q.

The interpretation of the anchor SNPs assumption is that every ancestral population has at least one SNP that appears only in it. The presence of such an SNP is therefore a guarantee that the individual is a member of a particular population. Note that an identical argument could be made when we have a set *S* of *d* columns of Q that have exactly one nonzero entry at unique locations. When such a set exists, we say that we have “*anchor individuals*.” Under the admixture model, the simplex constraint requires that the nonzero entry of each anchor genotype is exactly one. In this scenario, there exists at least one individual from each ancestral population whose entire genome was inherited by a single ancestral population. We summarize these results in the following corollary and visualize the anchor SNP and anchor genotype scenarios in Figure 1.

#### Corollary 1.

*Whenever a rank d matrix F admits a factorization F=PQ such that there are either a set of anchor SNPs or a set of anchor genotypes*, *the factorization is unique up to permutation*.

## Appendix B: tALS and cALS Comparisons

Figure 9 displays the $\hat{Q}$ estimates of the tALS and cALS algorithms on simulated data from each of the four α-prototypes described in *Simulated data sets*. For each of these datasets, $m=10^{5}$, n=500, d=3. We see that estimates provided by the tALS and cALS algorithms agree very well with each other for all α-prototypes. However, the run times are substantially different between these two methods, as displayed in Table 4: tALS terminates in minutes while cALS terminates in hours. Notably, the run times of the tALS algorithm also appear to be less sensitive to the α-prototype than the cALS algorithm. Most notably, the cALS algorithm takes an order of magnitude longer to run on the $\alpha_{4}$ prototype than any of the other α-prototypes. These observations support our preference for the tALS algorithm over the cALS algorithm.

Under $\alpha_{2}$ and $\alpha_{3}$, the estimates provided by tALS and cALS also agree very well with the true Q matrices. This is not the case under $\alpha_{1}$ and $\alpha_{4}$, where both algorithms provide substantially different results than the ground truth. However, because both of these α-prototypes lack a complete set of anchor SNPs, the model may well be unidentifiable.

## Appendix C: Simulation Details

Due to time and computational constraints, each algorithm did not terminate on each of the 96 datasets generated for the simulations. In all, 326 of the 384 total simulations terminated during the 1 week time limit with a budget of 300 GB. Admixture completed 81 simulations, fastSTRUCTURE completed 80 simulations terastructure completed 81 simulations, and ALStructure completed 84 simulations. For the sake of comparison, Figure 5 only shows the datasets for which all algorithms terminated.

## Appendix D: HGDP, TGP, and HO Dataset Details

In *Applications to Global Human Studies* we analyze human genotype data from globally sampled individuals. These data come from three public sources: HGDP (Cavalli-Sforza 2005), TGP (The 1000 Genomes Project Consortium *et al.* 2015), and HO (Lazaridis *et al.* 2014). The various preprocessing steps are detailed below for each dataset.

### TGP:

The 1000 Genomes Project dataset (TGP) samples globally from 26 populations and is available here: ftp://ftp.1000genomes.ebi.ac.uk/vol1/ftp/release/20130502/supporting/hd_genotype_chip/. Related individuals and SNPs with minor allele frequency <5% are removed. The dimensions of this dataset are 1716 individuals and 520,036 SNPs.

### HGDP:

The HGDP samples globally from 51 populations and is available here: http://www.hagsc.org/hgdp/files.html. Individuals with first- or second-degree relatives and SNPs with minor allele frequency <5% are removed. The dimensions of this dataset are 940 individuals and 550,303 SNPs.

### HO:

The Affymetrix HO dataset samples globally from 147 populations and is available here: http://genetics.med.harvard.edu/reich/Reich_Lab/Datasets.html. Nonhuman or ancient samples and SNPs with <5% minor allele frequency are removed. The dimensions of this dataset are 2248 individuals and 372,446 SNPs.

## Appendix E: Application to a Nonglobal Dataset

In this appendix we apply ALStructure and preexisting methods to a dataset from Basu *et al.* (2016). In this dataset, individuals from 18 mainland Indian subpopulations are sampled. Following Basu *et al.* (2016), we set d=4 for each method. Figure 10 plots the first two rows of Q output from Admixture, fastSTRUCTURE, terastructure, and ALStructure, respectively. As in the results from *Applications to Global Human Studies*, rows of Q are ordered according to variation explained.

As can be seen, the estimated admixture proportions produced by each method are all qualitatively similar. Table 5 shows the likelihood of the data from each method, with each method performing similarly. The methods ranked by decreasing mean log-likelihood are: Admixture, ALStructure, fastSTRUCTURE, terastructure.

**Table 4:** RMSE between true and estimated Q matrices for each method and each α-prototype (rows 1 and 2); RMSE between two estimated Q matrices (row 3); run-time (rows 4 and 5).

	$\alpha_{1}$	$\alpha_{2}$	$\alpha_{3}$	$\alpha_{4}$
$\text{RMSE}\left(Q,{\hat{Q}}_{\text{tALS}}\right)$	$6.2\times 10^{-2}$	$1.4\times 10^{-2}$	$8.7\times 10^{-3}$	$1.9\times 10^{-1}$
$\text{RMSE}\left(Q,{\hat{Q}}_{\text{cALS}}\right)$	$6.3\times 10^{-2}$	$1.5\times 10^{-2}$	$8.5\times 10^{-3}$	$2.2\times 10^{-1}$
$\text{RMSE}\left({\hat{Q}}_{\text{tALS}},{\hat{Q}}_{\text{tALS}}\right)$	$4.2\times 10^{-3}$	$2.3\times 10^{-3}$	$5.7\times 10^{-4}$	$3.9\times 10^{-2}$
tALS run time	2.6 min	1.4 min	1.5 min	2.8 min
cALS run time	4.1 hr	3.0 hr	3.5 hr	36.8 hr

**Table 5:** Mean log-likelihood from of each method applied to data set Basu *et al.* (2016)

Method	Admixture	fastSTRUCTURE	terastructure	ALStructure
Mean log-likelihood	−0.7360	−0.7369	−0.7373	−0.7365
